# Sequence Analysis of the Fusion Protein Gene of Human Respiratory Syncytial Virus Circulating in China from 2003 to 2014

**DOI:** 10.1038/s41598-018-35894-3

**Published:** 2018-12-04

**Authors:** Jinhua Song, Huiling Wang, Teresa I. Ng, Aili Cui, Shuangli Zhu, Yanzhi Huang, Liwei Sun, Zifeng Yang, Deshan Yu, Pengbo Yu, Hong Zhang, Yan Zhang, Wenbo Xu

**Affiliations:** 1 WHO WPRO Regional Reference Measles/Rubella Laboratory and Key Laboratory of Medical Virology, National Health Commission of the People’s Republic of China, National Institute for Viral Disease Control and Prevention, China Center for Disease Control and Prevention, Beijing, People’s Republic of China; 2 AbbVie, Inc, North Chicago, IL USA; 3 Jilin Children’s Medical Center, Children’s Hospital of Changchun, Changchun, People’s Republic of China; 4 State Key Laboratory of Respiratory Disease, National Clinical Research Center for Respiratory Disease, First Affiliated Hospital of Guangzhou Medical University, Guangzhou, Guangdong, People’s Republic of China; 5 Gansu Provincial Centers for Disease Control and Prevention, Lanzhou, People’s Republic of China; 6 Shaanxi Provincial Centers for Disease Control and Prevention, Xian, People’s Republic of China; 7 Hunan Provincial Centers for Disease Control and Prevention, Changsha, People’s Republic of China

## Abstract

The human respiratory syncytial virus (HRSV) fusion (F) protein is important for HRSV infection, but few studies have examined the genetic diversity of the F gene from Chinese samples. In this study, a total of 330 HRSV F sequences collected from different regions of China between 2003 and 2014 were analyzed to understand their genetic characteristics. In addition, these sequences were compared with 1150 HRSV F sequences in Genbank from 18 other countries. In phylogenetic analysis, Chinese HRSV F sequences sorted into a number of clusters containing sequences from China as well as other countries. F sequences from different genotypes (as determined based on the G gene sequences) within a HRSV subgroup could be found in the same clusters in phylogenetic trees generated based on F gene sequences. Amino acid analysis showed that HRSV F sequences from China and other countries were highly conserved. Of interest, F protein sequences from all Chinese samples were completely conserved at the palivizumab binding site, thus predicting the susceptibility of these strains to this neutralizing antibody. In conclusion, HRSV F sequences from China between 2003 and 2014, similar to those from other countries, were highly conserved.

## Introduction

Human respiratory syncytial virus (HRSV) is one of the leading pathogens causing lower-respiratory tract infections in infants and young children worldwide^1,2^. HRSV is a single-stranded, negative-sense RNA virus in the *Pneumoviridae* family. The attachment glycoprotein (G protein) and the fusion glycoprotein (F protein) are the two major glycoproteins on the HRSV surface. The G protein mediates the viral attachment to the host cells whereas the F protein mediates viral penetration and fusion of the infected cells^3–5^. HRSV could be divided into two subgroups, subgroup A (HRSVA) and subgroup B (HRSVB), based on the antigenic characteristics and the reactivity with monoclonal antibodies^6^. The HRSV G gene sequence is highly variable. Based on the sequences of the second hypervariable region of the G gene, HRSV strains from each subgroup are further classified into different genotypes. To date, 15 genotypes of HRSVA have been identified (GA1~7, NA1~4, ON1~2, SAA1, CBA)^7,8^ whereas 30 genotypes of HRSVB have been identified (GB1~4, BA1~14, BAc, SAB1~4, URU1~2, CB1(GB5), CBB, BA-CCA, BA-CCB and THB)^9–14^. According to the phylogenetic analysis of the G gene, the same predominant clades of HRSV circulated globally, and when different HRSV strains emerged, the distribution of the old clades could be changed^15^.

The F protein is synthesized as a precursor F0 protein [574 amino acids (aa) in length]. When the F0 protein passes through the Golgi, it can be activated by the cleavage with a furin-like intracellular host protease at 2 sites after amino acid residues 109 and 136 to generate three polypeptides: F1 (aa 137–574), F2 (aa 1–109) subunits and an intervening 27 amino acid peptide, pep27, (aa 110–136)^16,17^. The mature F protein is a homotrimer of the F1 and F2 subunits, and the F1 subunit is essential for the protein to cause membrane fusion. The F0 precursor contains 5 or 6 predicted N-linked glycosylation sites depending on the HRSV strain. After activation, 2 predicted N-linked glycosylation sites in F2, 1 predicted N-linked glycosylation site in F1 and 2–3 predicted N-linked glycosylation sites in in the pep27 are left^18,19^.

The F protein has been identified as having at least two dominant conformations: the prefusion and postfusion forms^20^. The functional F protein trimer in the virion membrane is in a metastable, prefusion form. This prefusion F protein had a ‘lollipop’ shape by electron microscopy^21,22^. In the prefusion form of the F1 protein, the fusion peptide at the N terminus of F1 is followed by 4 short α-helices connected by 3 non-helical peptides^5^. The structure of the postfusion F protein revealed a cone-shaped molecule, with a globular head and an extended stalk^21^. Three F2/F1 subunits that make up the trimeric molecule are tightly intertwined, with 3-fold symmetry that runs the length of the molecule. The globular head contains both the F2 and F1 subunits, as well as the cysteine-rich region. The stalk region is almost entirely helical, composed of the 6-helix bundle that is characteristic of the postfusion state of many type I viral fusion proteins^5,21,23^. The F protein is a target of virus-specific cytotoxic T lymphocytes (CTLs). Three related human HLA class I-restricted epitopes, HLA-A*01, HLA-B*57 and HLA-Cw*12, have been identified^24–26^, and 4 peptides of HRSVB were found to bind to HLA-A*0201 in HLA-A2 transgenic mouse^27^. In addition, the F protein is a target of neutralizing antibody and vaccine development due to its high sequence conservation. To date, 6 antigenic sites have been identified in F protein: Ø, I, II, IV, V, and VI. Antigenic sites I, II and IV are present in the postfusion F conformations^23,28^, whereas antigenic site Ø is found only in the prefusion F conformation^21^. Antigen site Ø is at the apex of the prefusion trimer composed of an α-helix from F1 and a strand from F2; the other 5 antigenic sites are all within the F1 subunit^5,21^. Antigenic site I is located in the middle of cysteine rich region^29^, and antigenic sites IV, V, and VI overlap with the C terminus of the cysteine rich region^30^. Antigenic site II is the binding site of the neutralizing antibodies palivizumab and motavizumab, especially the domain spanning aa 262–275^31–33^. It has been shown that mutations at some of the residues in this domain resulted in resistance to palivizumab and/or motavizumab^31,34,35^.

The molecular epidemiology of HRSV in China has been studied quite extensively based on the G gene^12,13^, but information regarding the genetic diversity and sequence characteristics of the F gene from Chinese HRSV samples is limited. In the present study, the phylogenetic relationship and the sequence diversity of the full-length coding DNA sequences (CDS) of the F genes from HRSV samples collected from different regions in China were compared to those from other countries.

## Results

### Samples information

A total of 181 (91 of HRSVA and 90 of HRSVB) HRSV samples were selected for F gene sequence analysis from over 700 HRSV-positive samples collected in 6 representative geographic regions of China between 2004 and 2014 (Supplementary Table S1). These samples were selected on the basis of their representation of the circulating strains in China [based on HRSV subgroup, genotype, genetic diversity (different clustering within a genotype in phylogenetic analysis using the sequences of the G gene), geographical region, and year of collection]^7,10^. In addition, all Chinese HRSV F gene sequences with full-length CDS that were available in GenBank as of October, 2016 (n = 149) (Supplementary Table S2) were downloaded and analyzed together with the 181 F sequences.

**Figure 1:**
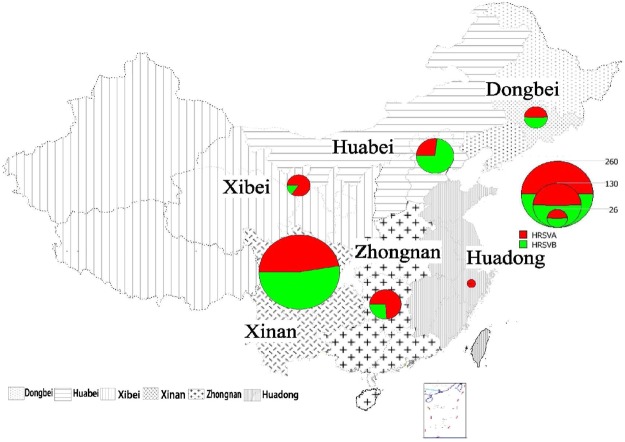
Geographic distribution of 330 HRSV F gene sequences in China from 2003 to 2014. The pie map of the number of China HRSV subgroup A (red) and B (green) sequences was generated using ArcGis software (version 10.2). Phylogenetic clusters of HRSVA and HRSVB are shown in Figs 2 and 3.

Therefore, a total of 330 Chinese HRSV F gene sequences collected from 2003 to 2014 were analyzed to determine their genetic diversity and sequence characteristics (Table 1 and Fig. 1). Moreover, 1150 HRSV F gene sequences (770 of HRSVA and 380 of HRSVB) with full-length CDS collected from 18 other countries between 1956 and 2014 were downloaded from GenBank for comparison study (Supplementary Table S2). More details of the sequences are available in Supplementary Tables S1,S2 and S3.

**Table 1:** Distribution of Chinese HRSV samples by geographical region and year.

Region	Dongbei	Huabei		Huadong	Xibei		Zhongnan		Xinan	Total
Province/City	Jilin	Beijing	Hebei	Shanghai	Gansu	Shaanxi	Hunan	Guangdong	Chongqing	
2003	—	—	3(0)	—	—	—	—	—	—	3(0)
2004	—	6(6)	—	—	—	—	—	—	—	6(6)
2008	—	5(5)	—	—	—	—	—	5(0)	—	10(5)
2009	1(1)	26(26)	—	—	—	—	—	2(2)	2(0)	31(29)
2010	—	9(9)	1(1)	4(4)	5(5)	5(5)	—	11(11)	18(0)	53(35)
2011	—	1(1)	—	—	8(8)	3(3)	1(1)	28(27)	81(0)	122(40)
2012	7(7)	—	—	—	—	—	5(5)	2(1)	31(0)	45(13)
2013	8(8)	—	4(4)	—	—	5(5)	8(8)	1(0)	5(0)	31(25)
2014	12(12)	10(10)	2(2)	1(0)	—	—	4(4)	—	—	29(29)
Total	28(28)	57(57)	10(10)	5(4)	13(13)	13(13)	18(18)	49(41)	137(0)	330(181)

Numbers shown are the sum of sequences collected in this study and those downloaded from GenBank; sequences collected in this study are shown within parentheses.

### Phylogenetic analysis of the F gene sequences

**Figure 2:**
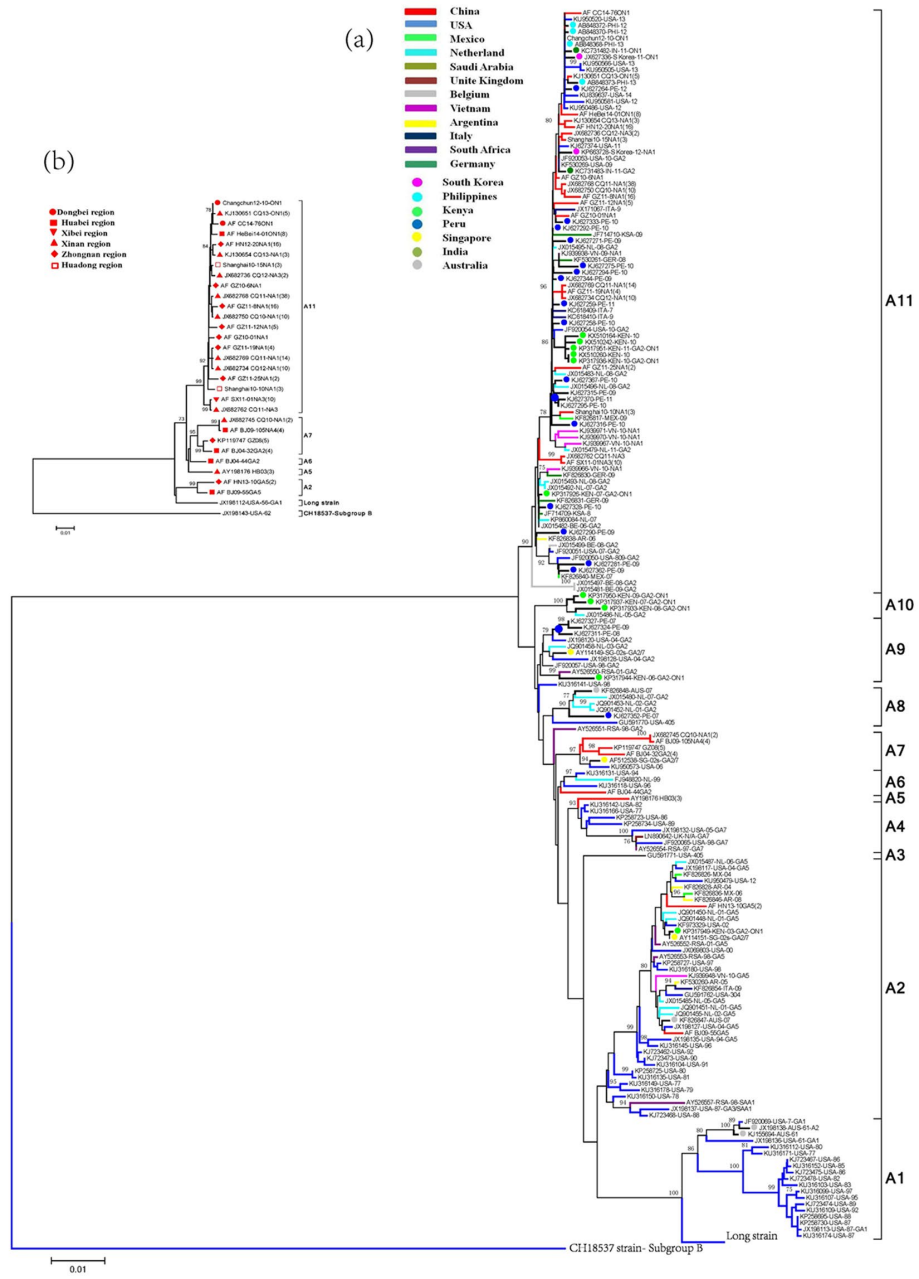
Phylogenetic trees of representative HRSVA F gene sequences from worldwide samples (**a**) and Chinese samples (**b**) from 1956 to 2014. Phylogenetic trees were generated using the neighbor joining method. Chinese sequences are denoted in red. In Panel b, the number within the parentheses behind the name of each representative sequence indicated the total number of sequences identical or similar (nucleotide difference less than 2) to that representative sequence.

A total of 986 full-length CDS of the HRSVA F gene, including 216 sequences from China, sorted to 11 clusters in phylogenetic trees generated by neighbor joining method, A1-A11 (Fig. 2). Among these sequences, Chinese HRSVA sequences sorted to 5 of these clusters: A2, A5, A6, A7 and A11 (Fig. 2a).

Genotype GA5 sequences from the Huabei and Zhongnan regions of China from 2008–2009 and 2013, and genotype SAA1 and GA5 sequences from the USA, the Netherlands, South Africa sorted into cluster A2. Three sequences from the Huabei region of China from 2003 sorted to cluster A5 with 93% bootstrap value. Sequences from the Huabei, Zhongnan and Xinan regions of China from 2004, 2008–2012, which were classified as GA2, NA1 or NA4 genotypes sorted to cluster A7 with 97% bootstrap value. Cluster A11, the largest group, was populated by Chinese sequences classified as genotypes NA1, NA3, or ON1 from 2010 to 2014, and genotype GA2, NA1 or ON1 sequences from Vietnam, USA, Kenya, the Philippines, South Korea and other countries from 2006–2014 with 90% bootstrap value. Most Chinese HRSVA sequences were in phylogenetic clusters containing sequences from China as well as other countries, but not in clusters that were solely made up of Chinese sequences (Fig. 2 and Supplementary table S4a).

**Figure 3:**
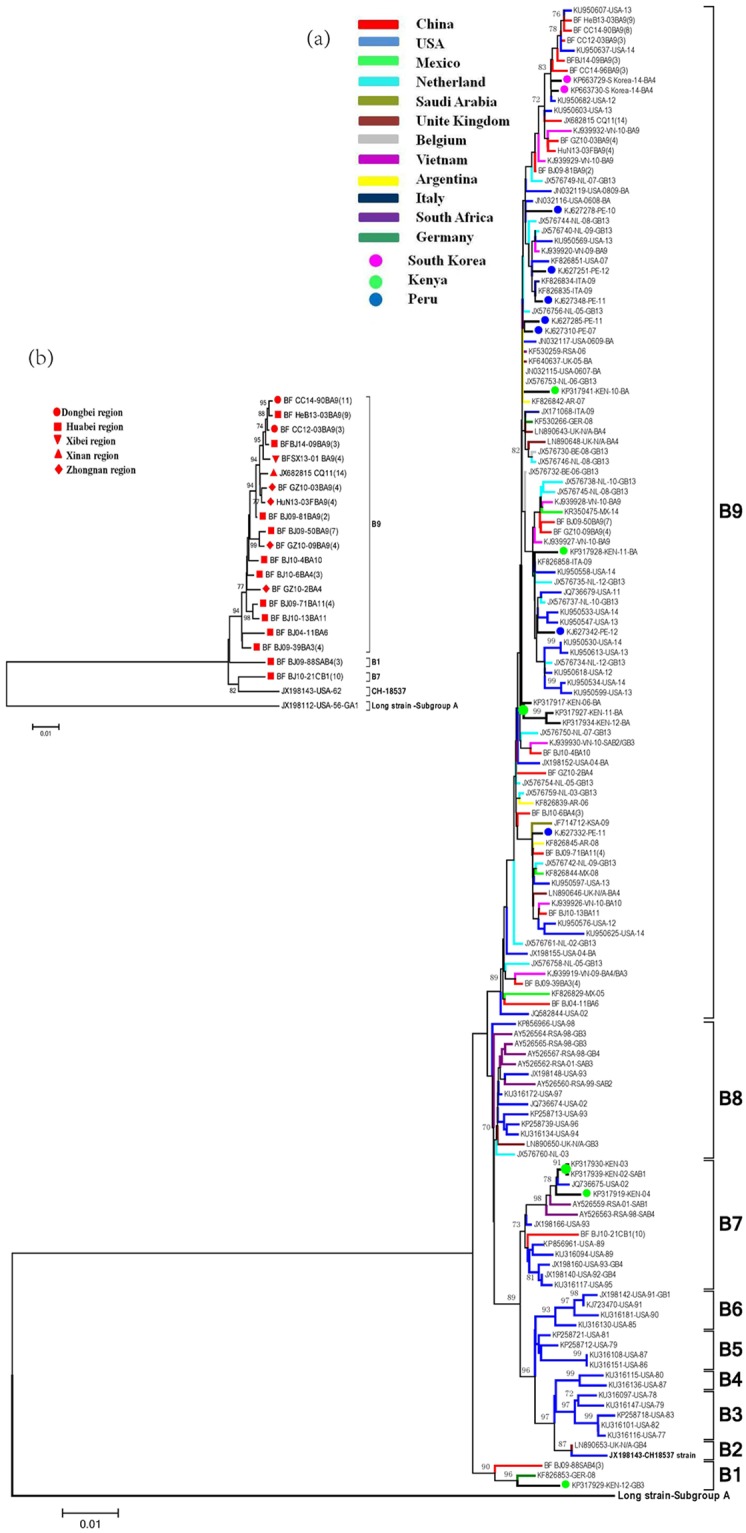
Phylogenetic trees of representative HRSVB F gene sequences from worldwide samples (**a**) and Chinese samples (**b**) from 1962 to 2014. Phylogenetic trees were generated using the neighbor joining method. Chinese sequences are denoted in red. In Panel b, the number within the parentheses behind the name of each representative sequence indicated the total number of sequences identical or similar (nucleotide difference less than 2) to that representative sequence.

A total of 494 full-length CDS of the HRSVB F gene, including 114 sequences from China, sorted to 9 clusters in phylogenetic trees generated by neighbor joining method, B1-B9 (Fig. 3). Chinese HRSVB sequences distributed into clusters B1, B7, and B9 (Fig. 3a).

Genotype CB1 sequences from the Huabei, Dongbei, Zhongnan and Xinan regions of China from 2009–2012, and sequences from the USA from 1989–1995 (GB4 genotype), Kenya from 2002–2004 (SAB1 genotype) and South Africa from 1998–2001 (SAB4 genotype) sorted to cluster B7 with 73% bootstrap value. Three SAB4 sequences from the Huabei region of China from 2009, 1 sequence from Germany in 2008 and 3 genotype GB3 sequences from Kenya from 2012 sorted to cluster B1 with 90% bootstrap value. Cluster B9, the largest group in the phylogenetic tree with 89% bootstrap value, contained mostly genotype BA sequences from different countries, including the USA, Netherlands, Belgium, Peru, Vietnam, Kenya, Italy, Germany and a large number of sequences from China from 2002 to 2014. Like the Chinese HRSVA sequences, Chinese HRSVB sequences were in phylogenetic clusters populated by sequences from China as well as the other countries (Fig. 3 and Supplementary table S4b).

The phylogenetic analyses were also conducted using the maximum likelihood method. Phylogenetic trees generated by maximum likelihood method (Supplementary Figures S1 and S2) had the same cluster designations.

### Pairwise-distance calculations of the F sequences

To determine the sequence variability of the F genes from samples collected in China or other countries, the p-distances of the sequences in the nucleotide and deduced amino acid levels were calculated. Overall, nucleotide and amino acid p-distances of the F sequences within the HRSVA or HRSVB subgroup from different countries were much smaller than those between the 2 subgroups within the same country (Table 2), indicating that the sequences were more similar within a subgroup than between subgroups. The nucleotide and amino acid p-distances of F sequences from China within or between the HRSVA or HRSVB subgroups were similar to those values for other countries. The nucleotide p-distances were usually ≤0.03 or ≤0.02 within the HRSVA or HRSVB subgroup, respectively, whereas the amino acid p-distances were usually ≤0.01 or ≤0.007 within the HRSVA or HRSVB subgroup, respectively. In contrast, the nucleotide and amino acid p-distances between the HRSVA and HRSVB F sequences from each of the source countries were in the range of 0.20–0.211 and 0.093–0.104, respectively. Furthermore, the nucleotide p-distance between each cluster in a HRSVA or HRSVB phylogenetic tree was in the range of 0.02–0.06 or 0.01–0.03, respectively (Supplementary Tables S5a and S5b).

**Table 2:** Nucleotide and amino acid p-distances within/between HRSVA and HRSVB F sequences.

Country^a^	p-distance					
	Within HRSVA		Within HRSVB		Between HRSVA and HRSVB	
	Nucleotide	Amino acid	Nucleotide	Amino acid	Nucleotide	Amino acid
AR	0.017	0.007	0.007	0.005	0.202	0.103
AUS	0.037	0.022	—	—	—	—
BE	0.008	0.003	0.002	0.003	0.207	0.093
CHN	0.01	0.005	0.013	0.007	0.208	0.1
GER	0.009	0.007	0.022	0.009	0.211	0.102
IN	0.003	0.005	—	—	—	—
ITA	0.024	0.011	0.004	0.003	0.207	0.101
KEN	0.005	0.004	0.017	0.007	0.21	0.103
KSA	0.011	0.007	—	—	0.207	0.101
MEX	0.021	0.009	0.013	0.006	0.204	0.103
NL	0.023	0.011	0.02	0.01	0.2	0.098
PE	0.01	0.005	0.007	0.003	0.209	0.098
PHI	0.001	0.002	—	—	—	—
RSA	0.024	0.007	0.012	0.006	0.204	0.099
SK	0.005	0.007	0.003	0.002	0.206	0.104
SG	0.024	0.016	—	—	—	—
UK	—	—	0.016	0.011	0.201	0.099
USA	0.029	0.011	0.018	0.007	0.208	0.1
VN	0.01	0.004	0.007	0.005	0.209	0.1

a: Abbreviations of countries: AR = Argentina, AUS = Australia, BE = Belgium, CHN = China, GER = Germany, IN = India, ITA = Italy, KEN = Kenya, KSA = Saudi Arabia, MX = Mexico, NL = Netherlands, PE = Peru, PHI = Philippines, RSA = South Africa, SK = South Korea, SG = Singapore, UK = United Kingdom, USA = America, VN = Vietnam.

Of interest, F sequences of HRSVA appeared to be slightly more variable than those of HRSVB at both the nucleotide and amino acid levels (Table 2). When the amino acid sequences of the F protein from a country were compared to those from another country, there was usually over 98% or 99% identity for HRSVA or HRSVB sequences, respectively (Supplementary Table S5c and d). HRSV F sequences from China and other countries were highly conserved at both the nucleotide and amino acid levels.

### Sequence analysis of the neutralizing epitopes

The F protein harbors epitopes targeted by RSV-neutralizing antibodies. To analyze the sequence variation due to natural polymorphisms at the neutralizing epitopes of Chinese HRSV F protein sequences in comparison with those from other countries, all HRSVA (216 from China and 770 from other countries) and HRSVB (114 from China and 380 from other countries) F protein sequences were aligned with the sequences of the reference strains Long and CH18537, respectively.

All of the 216 Chinese HRSVA F sequences were 100% conserved at antigenic site Ø first domain [aa 62–69], and antigenic site II (aa 255–275), whereas 1 out of the 216 Chinese HRSVA F sequences had a substitution (L203F) at antigenic site Ø second domain (aa 196–210) compared to the reference sequence of Long (Supplementary Table S6). At antigenic site I (aa 380–400), V384I/T substitutions were observed in 947 (96%) HRSVA sequences from China and other 18 countries collected during the past 40 years. Substitutions V384I/T were only found in HRSVA sequences from Chongqing city in the Xinan region of China from 2011. Most of the other substitutions were less common. Substitution N380S was observed in 4 sequences collected in 2008 from Guangdong province in the Huanan region of China. Substitutions L381F, I395V and S436Y were each observed in 1 sequence from China.

For HRSVB F sequences, all Chinese sequences were 100% conserved at antigenic sites I, IV, V and VI (Supplementary Table S7). A number of HRSVB F sequence variations were identified at antigenic site Ø, e.g. 8 sequences from China (3 sequences of the SAB4 genotype from Beijing), Germany, Kenya, the UK and the USA had K65Q/T, 492 (~100%) sequences from different countries (including China) harbored the substitution R202Q, and 61 sequences from China and the USA carried Q209K/R. For antigenic site II (aa 255–275), 1 Chinese sequence from Beijing was found to have S259A, while 1 sequence from the USA had S255G. Overall, the sequences at the antigen site II of the F protein from HRSVA and HRSVB samples collected globally, including those from China, were highly conserved.

Over 95% of sequences had substitutions at a number of amino acid positions located outside of the antigenic sites: amino acid positions 8, 20, 101, 124, 213 and 515 in HRSVA (Supplementary Table S6), and positions 17, 152, 185 and 202 in HRSVB (Supplementary Table S7). Substitution R213S was detected in 981 (99%) HRSVA sequences from 17 countries from 1961–2014, including all (216) HRSVA sequences from China.

### Sequence analysis of the cytotoxic T lymphocyte epitopes

Virus-specific CTLs play an important role in the clearance of HRSV infection. To date, 4 CTL epitopes have been identified in the F protein: HLA-A*01 (aa 109–118), HLA-B*57 (aa 106–114), HLA-Cw*12 (aa 551–559) and HLA-A*0201 (aa 33–41, 214–222, 273–281 and 559–567).

In this study, no substitutions were detected at aa 106, 108–118 and 551–559 in HRSVA sequences from China. Two Chinese sequences had a substitution at aa 107 (A107T) (Supplementary Table S6). For HRSVB F protein sequences, aa 108–111, 116, 118, and 555–558 were 100% conserved worldwide (Supplementary Table S7). Substitutions N554K/S/T were observed in 5 Chinese HRSVB sequences, and 1 HRSVB sequence from Beijing China had the L559I substitution. HRSVA N276S substitution was observed in 84.3% (182/216) Chinese sequences and 57.3% (441/770) sequences from other countries in 2007–2014, whereas only 4 HRSVB sequences from China (n = 1) and the USA (n = 3) collected in 2013–2014 had a substitution at this amino acid position (S276N). One Chinese HRSVB sequence contained I221V.

Overall, CTL-specific epitopes in HRSV sequences from all countries, including China, were well conserved. The substitutions, if any, in the sequence of the epitopes appeared to vary by positions depending on the countries of origin of the samples, but some substitutions (e.g. N276S in HRSVA) were found across different countries.

### Sequence analysis of the N/O-glycosylation sites of Chinese HRSV F sequences

N- or O-glycosylation can modify the biological activity of a protein. In this study, 6 N-glycosylation sites (aa 27, 70, 116, 120, 126 and 500) were predicted in the F protein of the HRSV reference strains of Long and CH18537 (Table 3). All Chinese F protein sequences had the 6 predicted N-glycosylation sites, with the exception of a single HRSVA sequence from Guangdong which had a mutation at aa 122 (T122P) leading to the change of the amino acid sequence from NNTK to NNPK and the loss of the predicted N-glycosylation site at aa position 120. In contrast to the prediction of N-glycosylation sites in almost all HRSV sequences, O-glycosylation sites were predicted in 42 HRSV sequences by the NetOGlyc 4.0 method: 6 O-glycosylation sites (aa 99, 100, 101, 118, 128 and 244) were predicted in 20 Chinese HRSVA F sequences and 8 O-glycosylation sites (aa 100, 102, 105, 115, 118, 122, 128 and 130) were predicted in 22 Chinese HRSVB F sequences. The more common amino acid positions with predicted O-glycosylation sites in HRSVA or HRSVB F sequences included positions 99, 118 and 128. The O-glycosylation site at aa 128 was predicted in 19 HRSVB sequences and 2 HRSVA sequences from the Xinan, Zhongnan and Huabei regions in China. O-glycosylation at these predicted sites needs to be investigated, as O-linked glycan have not been reported on a HRSV F protein previously.

**Table 3:** Predicted N/O-glycosylation sites in Chinese HRSV F sequences.

Site	Sequence name or accession numbers	Amino acid	Subgroup
**N-glycosylation**			
NITE	All Chinese sequences	27	A/B
NGTD	All Chinese sequences	70	A/B
NYTL	All Chinese sequences	116	A/B
NNTK	All Chinese sequences except Guangzhou10–01(HRSVA)	120	A/B
NVTL	All Chinese sequences	126	A/B
NQSL	All Chinese sequences	500	A/B
**O-glycosylation**			
99S	BJ04–34, BJ09–105, KP119745 GZ11–161, BJ04–23, JX682745, BJ04–44, GZ11–21, JX682724, GZ11–18, KP119748	99	A
100T	BJ04–34, BJ09–105, BJ04–23, KP218910, BJ04–32, GZ11–18	100	A
101S	BJ10–2, JX682716, BJ04–01, JX682745	101	A
118T	BJ04–34, BJ09–105, KP119745, GZ11–16, BJ04–23, KP218910, KP119746, BJ10–2, JX682716, BJ04–01, GZ11–21, JX682724, GZ11–18, KP119748	118	A
128T	GZ11–16, JX682724	128	A
244T	GZ10–01, JX682718	244	A
100T	GZ09–1	100	B
102S	GZ09–1	102	B
105S	BJ09–43	105	B
115T	CC14–49	115	B
118T	CC14–49	118	B
122T	GZ10–05, GZ10–04, BJ09–112, HN14–16, BJ09–21, BJ10–20	122	B
128S	GZ10–05, JX682801, BJ09–75, GZ10–09, JX682807, BJ09–64, JX682820, BJ09–110, JX682808, GZ10–04, HN12–30, BJ10–16, BJ09–112, HN14–16, JX682803, BJ09–21, BJ09–50, BJ09–65, BJ10–20	128	B
130S	HN12–30	130	B

### Selection pressure site prediction

The selection pressure on Chinese HRSV was estimated using the dN/dS ratio. The mean dN/dS ratios for Chinese HRSVA and HRSVB were 0.092 and 0.098, respectively, while the mean dN/dS ratios for HRSVA and HRSVB from other countries were 0.106 and 0.120, respectively (Table 4). In this analysis, dN/dS ratios > 1 were considered as evidence of positive selection. More negative selection sites than positive selection sites were predicted in HRSV sequences from China and other countries. No positive selective sites were detected by the single likelihood ancestor counting (SLAC) method in all HRSV F sequences from China or other countries, whereas a total of 3 positive selective sites (aa 125, 201, 574) in Chinese sequences and a total of 4 positive selective sites (aa 15, 152, 384, 573) in sequences from other countries were predicted by the fixed effects likelihood (FEL) and the internal fixed effects likelihood (IFEL) methods.

**Table 4:** Predicted selection pressure sites in HRSV F sequences.

Sequence Origin	HRSV subgroup	Mean *dN/dS*	No. of positive selective sites (aa position, p-value)			No. of negative selective sites			Significance *p-value*
			SLAC	FEL	IFEL	SLAC	FEL	IFEL	
China	A	0.092	0	0	1 (574, 0.049)	31	92	25	0.05
	B	0.098	0	1 (125, 0.035)	1 (201, 0.032)	21	54	8	0.05
Other countries	A	0.106	0	0	2 (152, 0.15; 384, 0.045)	159	234	148	0.05
	B	0.120	0	2 (15, 0.036; 573, 0.035)	0	70	141	284	0.05

SLAC = single likelihood ancestor counting method, FEL = fixed effects likelihood method, IFEL = internal fixed effects likelihood method.

## Discussion

Little is known about the sequence characteristics and genetic diversity of Chinese HRSV F gene. In this study, 330 Chinese HRSV F gene sequences from different regions in China were analyzed together with 1150 HRSV F gene sequences from 18 other countries by phylogenetic analysis and other sequence analyses. The present study provides important information regarding the genetic diversity and sequence characteristics of Chinese HRSV F sequences from 2003–2014. Results presented in this study provide information for the development of vaccines, neutralizing antibodies and other therapies for HRSV infection.

The sequence of the HRSV G gene is highly variable. Due to the genetic diversity in the G gene, sequence encoding the second hypervariable region in the C-terminal of the G protein has been used for genotyping of HRSV. In contrast, the sequence of the HRSV F gene is highly conserved. Most of the molecular epidemiology studies of HRSV were conducted using the sequences of the G gene. However, there is increased interest in the molecular epidemiology of the F protein in recent years as F protein is a target of neutralizing antibodies and vaccine development. In this study, phylogenetic analysis of ~1500 HRSV F sequences from samples collected from the extended period of 1956–2014 worldwide, including 330 sequences from China from 2003–2014, identified 11 and 9 clusters with the sequences of the HRSVA and HRSVB subgroups, respectively. Of interest, F sequences from different genotypes (as determined based on the G gene sequences) within a HRSV subgroup could be found in the same clusters in phylogenetic trees generated based on F gene sequences, e.g. cluster A11 of HRSVA contained sequences from HRSVA genotypes NA1, NA3 and ON1.

Our p-distance calculations showed that there was a high level of sequence identity between the F sequences from China and other countries. In addition, the variability of the F sequences from HRSVA was slightly higher than that from HRSVB samples collected from China as well as many other countries, which was consistent with observations reported previously^26^.

All Chinese HRSVA sequences were highly conserved at antigenic site Ø (aa 62–69 and 196–210), but 3 Chinese HRSVB sequences classified as the SAB4 genotype from Beijing had the K65Q substitution and sorted to a separate group in the B7 cluster of the phylogenetic tree, suggesting that K65Q could be a genotype specific amino acid change. Other HRSVB substitutions such as R202Q and Q209K/R were also detected at antigenic site Ø. Antigenic site I is located in the middle cluster of the cysteine-rich region of the F1 chain. The F1 subunit is essential for membrane fusion^19^. Antibodies binding to antigenic site I have marginal effect in virus neutralization. Similar to antigenic site I, antigenic sites IV, V, and VI are also located near the C-terminal end of the cysteine-rich region and not far from the heptad repeat adjacent to the membrane of the F1 chain. During the fusion process, neutralizing antibodies binding to both sides of the cysteine cluster would inhibit conformational changes of the F1 chain^30^. Previous studies have shown that substitutions at aa 262, 268, 272 and 275 of the F protein conferred resistance to palivizumab *in vitro* or *in vivo*^34,35^. Only 1 Chinese HRSVB sequence has the substitution of S259A at antigenic site II. The impact of the S259A substitution on viral pathogenesis remains to be determined.

The CTL epitopes were well conserved in HRSV sequences from China and other countries. However, N276S substitution was commonly detected in the CTL epitopes of HRSVA F sequences from China as well as many other countries. In our study, 84.3% (182/216) Chinese HRSVA sequences carried the N276S substitution. The percentage of Chinese HRSVA strains with the N276S substitution was found to be increasing after 2014^36,37^. Although aa N276 is next to the palivizumab binding site (aa 262–275), the N276S substitution has been proved to be a natural polymorphism and does not impact the susceptibility of HRSVA to palivizumab^35,37^.

N-glycans on viral glycoproteins are important structural components that could affect the folding, transport, activity, stability and immunological properties of the viral glycoproteins^19^. It has been shown that the loss of the N-glycan in HIV gp120 could affect the sensitivity of HIV to neutralizing antibodies and modulate the structure, stability or accessibility of viral epitopes in the CD4 binding site and coreceptor binding region of HIV^38^. There are generally 5 or 6 potential N-linked glycosylation sites in HRSV F protein depending on the viral strains^5^. In this study, only 1 out of 330 Chinese HRSV F sequences was found to have lost one of the N-glycosylation sites (aa 120), which is located in pep27 (aa 110–136); previous study has shown that pep27 has no impact for virus maturation and infectivity^39^. Substitutions at aa N500 resulted in pronounced reduction of the fusion activity of F protein by about 90~100%, indicating that the single N-glycan of the F1 subunit at aa N500 was essential for efficient syncytium formation. N-glycosylation of the F2 subunit at aa N27 and N70 have been shown to have minor impact on the fusion activity of the F protein^19^. In this study, a number of O-glycosylation sites were predicted in Chinese HRSV F protein sequences. This predication needs to be investigated further, as there has been no report of O-linked glycan on a HRSV F protein^19^.

Previous study showed that the dN/dS ratios for the hemagglutinin genes of wild-type measles virus H1 genotype were <1 and demonstrated the hemagglutinin amino acid substitutions were the result of random genetic drift rather than accumulated mutations^40^. In this study, we found that the mean dN/dS ratios of the F genes for both HRSVA and HRSVB strains in China and other countries had a value < 1, suggesting that the amino acid substitutions in F protein of HRSV were also the results from random genetic drift. In addition, it was reported that most HRSV genes, with the exception of the G gene, have negative selection or neutrally evolving sites^41^. Consistent with this finding, we identified only 3 positive selection sites for HRSVA (aa 152, 384 and 574) and 4 positive selection sites for HRSVB (aa 15, 125, 201 and 573) in ~1500 HRSV F sequences collected worldwide. Positive selection sites at aa 15 and aa 125 are located in the F2 subunit and pep27, respectively, whereas the other 5 positive selection sites are all located in the F1 subunit. Amino acid 384 is located in antigenic site I, and we found that the substitutions V384I/T were present in 96% HRSVA sequences collected all over the world. Amino acid 125 is located in pep27. Recently, it has been shown that the binding to a pep27-containing peptide (F 101–121) was higher with sera from HRSV-infected infants with neutralizing antibodies than infants without neutralizing antibodies, indicating the presence of novel antigenic site(s) in the unprocessed F0 conformation^42^. Consistent with the results from previous studies^20,36^, substitutions L125S/P were found in 8% HRSVB sequences in our study. The potential effect of substitutions at these positive selection sites on the pathogenesis of HRSV remains to be determined.

There are currently no specific antiviral treatments or vaccines approved for HRSV infection. Palivizumab is a humanized monoclonal antibody indicated for the prevention of serious lower respiratory tract disease caused by HRSV infection in high-risk infants in the USA and other countries. Palivizumab binds to the highly conserved antigenic site II of the HRSV F protein, and is cross-reactive with the F protein of HRSVA and HRSVB due to the sequence conservation at its binding site^32,43^. Only a small number of substitutions associated with resistance to palivizumab have been identified *in vitro* or *in vivo*; these substitutions were at aa 262, 268, 272 and 275 of the F protein^34,35^. In this study, we found that the palivizumab binding site in the F protein sequences from all of the 330 Chinese HRSV samples was 100% conserved, and 100% identical to the corresponding site found in the F protein sequences of prototypic HRSVA and HRSVB strains, both which are susceptible to the neutralization by palivizumab. No mutations were found at the amino acid positions which are known to confer resistance to palivizumab. Taken together, these results predict that the endemic Chinese HRSV strains would be susceptible to the neutralizing effect of palivizumab in the clinical setting. The susceptibility of Chinese HRSV strains to neutralization by palivizumab should be confirmed *in vitro* and *in vivo*.

There are some limitations in the present study. This study is a retrospective study and the source of available sequences was limited by the number of samples collected previously. Most of the sequences were downloaded from GenBank without the information of DNA sequencing methods, which might have different detection limits. In addition, while sequences of most samples were amplified directly from clinical specimens, some were amplified from samples previously isolated, which might have some impact on the sequencing results. Different countries had different number of sequences and the collection dates of the samples were not successive. Furthermore, samples were from 6 representative regions of China instead of from all over China, and the number of samples from each region was different.

In conclusion, this study investigated the sequence diversity of the HRSV F gene from 330 samples collected in China from 2003–2014. The F sequence was highly conserved in HRSV strains from China as well as those from 18 other countries. The high level of sequence conservation in the HRSV F sequences worldwide supports the F protein as a target for vaccine development and antiviral therapy. The evolution of the F gene of HRSV strains from China and the rest of the world should be monitored continuously for genetic diversity and changes in functional and antigenic properties.

## Methods

### Ethics statement

This study did not involve human experimentation and only nasopharyngeal precipitates (aspirates) were collected from patients with respiratory infections. Written informed consent for the use of their nasopharyngeal precipitates was obtained from adult patients and parents or guardians of pediatric patients. This study was approved by the second session of the Ethics Review Committee of the National Institute for Viral Disease Control and Prevention (NIVDC) of the Center for Disease Control and Prevention (CDC) in China and the methods were performed in accordance with the approved guidelines.

### Sample collection

181 representative Chinese HRSV samples were selected from over 700 HRSV-positive samples for the determination of the full-length CDS of the F genes based on HRSV subgroup, genotype (designation based on the sequence of the G gene), genetic diversity (different clustering within a genotype in phylogenetic analysis using the sequences of the G gene), geographical region, and year of collection^7,10^. The 181 samples included 91 HRSVA samples (40 isolates and 51 clinical specimens) and 90 HRSVB samples (18 isolates and 72 clinical specimens) from 5 representative geographic regions of China (Dongbei, Huabei, Huadong, Zhongnan and Xibei regions) between 2004 and 2014 (Supplementary Table S1). The genotypes of the samples included GA2, GA5, NA1, NA3, NA4, ON1, BA3, BA4, BA6, BA9, BA10, BA11, CB1 and SAB4 (genotypes determined based on the sequence of the second hypervariable region of the G gene as described in our previous studies^7,10^. The 181 Chinese F gene sequences were submitted to GenBank with the accession numbers of KY296617-KY296797, as shown in Supplementary Table S1.

### Viral RNA extraction

Viral RNA was extracted from clinical specimens or cultured isolates identified as positive of HRSV using the QIAamp RNA mini kit (QIAGEN, Valencia, CA, USA) according to the manufacturer’s instructions.

### Nucleotide amplification and sequencing

The second hypervariable region of the G gene of each HRSV sample was first sequenced and then analyzed in phylogenetic analysis to determine its genotype as described previously^7,10^. Depending on the designated genotypes and other criteria as described in “Sample collection” above, 181 HRSV samples were selected for F gene sequencing.

PCR amplifications of the full-length CDS of the F gene (in 3 separate fragments) were performed using the One Step RT-PCR kit (TaKaRa Biotechnology, Dalian, China). The PCR and sequencing primers are shown in Supplementary Table S8. Reaction mix contained 5 ul RNA, 12.5 ul reaction buffer, 1 ul One Step Enzyme Mix and 0.4 uM of a forward and reverse primer. The amplification was conducted at 50 ° for 30 min for reverse transcription, 94 °C for 2 min for denaturation, and with 40 cycles of 94 °C for 30 sec, 50 °C for 30 sec, 72 °C for 60 sec (105 sec for HRSV F2 fragment) for amplification, followed by a final extension at 72 °C for 10 min. The PCR products were all purified using a QIAquickGel Extraction Kit (Qiagen) and sequenced using an ABI Prism 3710 × 1 DNA Analyzer. The sequences were edited using Sequencher software vision 5.0 (Gene Codes, Ann Arbor, MI, USA).

### Phylogenetic, p-distance and amino acid variation analyses

Phylogenetic trees were generated using the software MEGA 5.0 with the neighbor joining method with Kimura 2-parameter model or maximum likelihood method with GTR + G + I model. Evaluation of the reliability of phylogenetic inference was estimated using the bootstrap method with 1000 replicates with a cut-off value of usually >70^44^. In this study, a cluster was defined as a group of sequences within a distinct branch in the phylogenetic tree, and the higher bootstrap, the higher reliability. Sequences were aligned using ClustalW in the MEGA 5.0 software and the pairwise distance (p-distance) of nucleotide and deduced amino acid among sequences were also calculated using MEGA 5.0^45^. The N- and O-glycosylation sites were predicted using the NetNGlyc 1.0 and NetOGlyc 4.0 server^46,47^, respectively. Selection pressure was determined on the Datamonkey website (http://www.datamonkey.org/) by estimating the ratio of non-synonymous (dN) and synonymous (dS) substitution per site based on the SLAC, FEL, and IFEL methods with a significance level of 0.05. The reference strains used in this study were Long (GenBank accession number JX198112) and CH18537 (GenBank accession number JX198143).

### Nucleotide sequence accession numbers and HRSV sequences downloaded from GenBank

All full-length HRSV F gene sequences that were available in GenBank as of October 2016, including 149 sequences from Xinan, Zhongnan, Huabei and Huadong regions of China and 1150 sequences from 18 other countries from Asia, Europe, North America, South America, Australia, and Africa (details shown in Supplementary Tables S2 and S3) were downloaded for analysis together with the 181 full-length F gene sequences that were generated from samples collected in this study.

## Electronic supplementary material

Supplementary figures

Dataset 1
