# Supplementary material for: Sequence Analysis of the Fusion Protein Gene of Human Respiratory Syncytial Virus Circulating in China from 2003 to 2014
Source: Sci Rep. 2018 Dec 4;8:17618. doi: 10.1038/s41598-018-35894-3 (PMC6279739; doi:10.1038/s41598-018-35894-3)
Supplement: Supplementary file 1 — Supplementary figures [file 41598_2018_35894_MOESM1_ESM.pdf]

Sequence Analysis of the Fusion Protein Gene of Human Respiratory Syncytial Virus Circulating in China from 2003 to 2014

Jinhua Song[1], Huiling Wang[1], Teresa I. Ng[2], Aili Cui[1], Shuangli Zhu[1], Yanzhi Huang[3], Liwei Sun[3], Zifeng Yang[4], Deshan Yu[5], Pengbo Yu[6]， Hong Zhang[7], Yan Zhang[1#], Wenbo Xu[1#]

[1]WHO WPRO Regional Reference Measles/Rubella Laboratory and Key Laboratory of Medical Virology, Ministry of Health, National Institute for Viral Disease Control and Prevention, China Center for Disease Control and Prevention, Beijing, People's Republic of China. [2]AbbVie, Inc., North Chicago, IL, USA. [3]Jilin Children's Medical Center, Children's Hospital of Changchun, Changchun, People's Republic of China.[4]State Key Laboratory of Respiratory Disease, National Clinical Research Center for Respiratory Disease, First Affiliated Hospital of Guangzhou Medical University, Guangzhou, Guangdong, People's Republic of China. [5]Gansu Provincial Centers for Disease Control and Prevention, Lanzhou, People's Republic of China. [6]Shaanxi Provincial Centers for Disease Control and Prevention, Xian, People's Republic of China. [7]Hunan Provincial Centers for Disease Control and Prevention, Changsha, People's Republic of China.

[#]Corresponding author: E-mail address: Yan Zhang, zhangyan9876543@163.com， Wenbo Xu, E-mail address: wenbo_xu1@aliyun.com

Supplementary Figure S1. Phylogenetic tree generated by maximum likelihood method of representative HRSVA F sequences from worldwide samples and Chinese samples from 1956 to 2014. For representative Chinese sequences that have sequences identical or similar (nucleotide difference less than 2) to them, the total numbers of identical or similar sequences are shown within parentheses behind the names of the respective representative sequences.

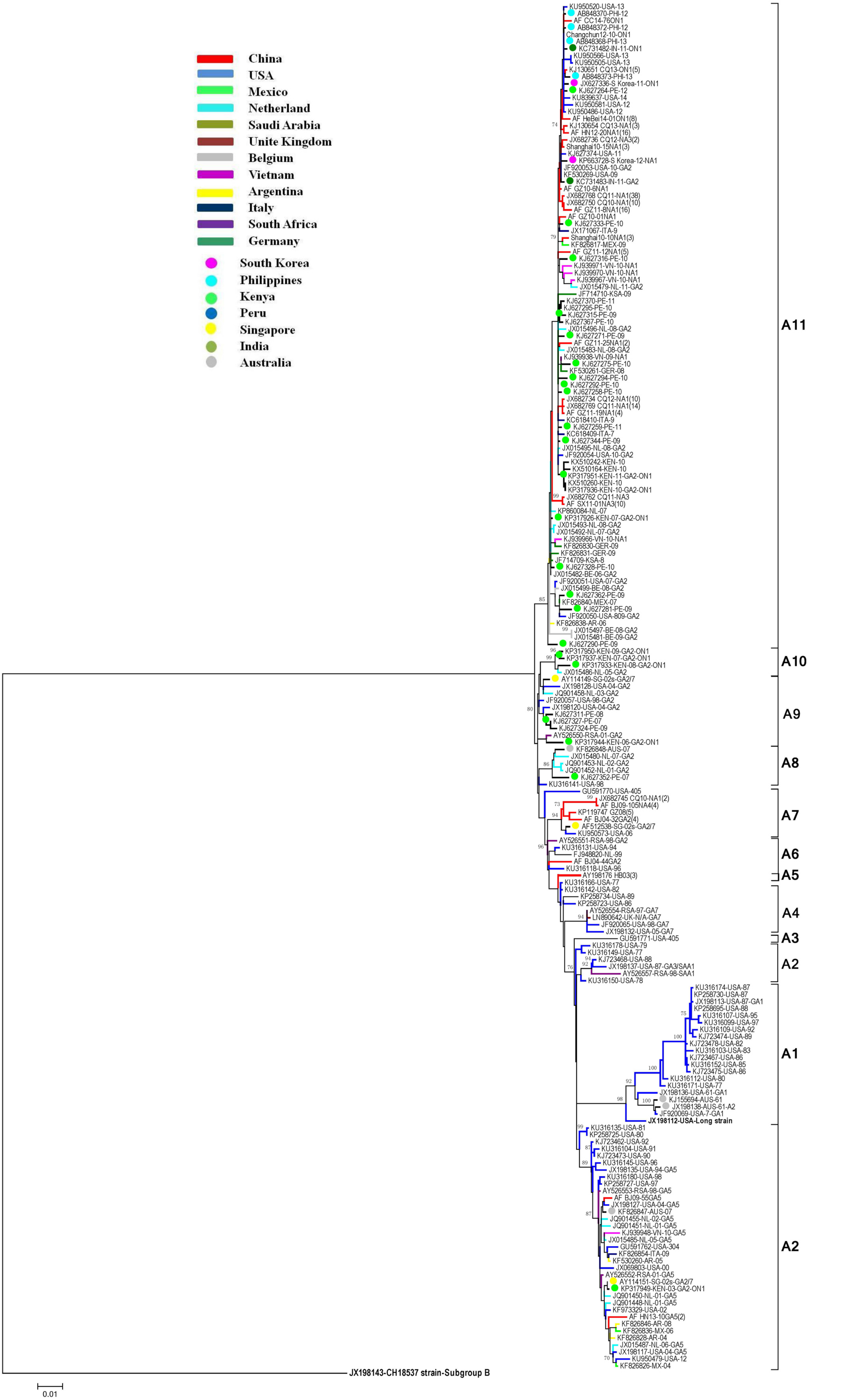
China
USA
Mexico
Netherland
Saudi Arabia
Unite Kingdom
Belgium
Vietnam
Argentina
Italy
South Africa
Germany
South Korea
Philippines
Kenya
Peru
Singapore
India
Australia
KU950520-USA-13
AB848370-PHI-12
AF CC14-76ON1
AB848372-PHI-12
Changchun12-10-ON1
AB848368-PHI-13
KC731482-IN-11-ON1
KU950566-USA-13
KU950505-USA-13
KJ130651 CQ13-ON1(5)
AB848373-PHI-13
JX627336-S Korea-11-ON1
KJ627264-PE-12
KU839637-USA-14
KU950581-USA-12
KU950486-USA-12
AF HeBei14-01ON1(8)
KJ130654 CQ13-NA1(3)
AF HN12-20NA1(16)
JX682736 CQ12-NA3(2)
Shanghai10-15NA1(3)
KJ627374-USA-11
KP663728-S Korea-12-NA1
JF920053-USA-10-GA2
KF530269-USA-09
KC731483-IN-11-GA2
AF GZ10-6NA1
JX682768 CQ11-NA1(38)
JX682750 CQ10-NA1(10)
AF GZ11-8NA1(16)
AF GZ10-01NA1
KJ627333-PE-10
JX171067-ITA-9
Shanghai10-10NA1(3)
KF826817-MEX-09
AF GZ11-12NA1(5)
KJ627316-PE-10
KJ939971-VN-10-NA1
KJ939970-VN-10-NA1
KJ939967-VN-10-NA1
JX015479-NL-11-GA2
JF714710-KSA-09
KJ627370-PE-11
KJ627295-PE-10
KJ627315-PE-09
KJ627367-PE-10
JX015496-NL-08-GA2
KJ627271-PE-09
AF GZ11-25NA1(2)
JX015483-NL-08-GA2
KJ939938-VN-09-NA1
KJ627275-PE-10
KF530261-GER-08
KJ627294-PE-10
KJ627292-PE-10
KJ627258-PE-10
JX682734 CQ12-NA1(10)
JX682769 CQ11-NA1(14)
AF GZ11-19NA1(4)
KC618410-ITA-9
KJ627259-PE-11
KC618409-ITA-7
KJ627344-PE-09
JX015495-NL-08-GA2
JF920054-USA-10-GA2
KX510242-KEN-10
KX510164-KEN-10
KP317951-KEN-11-GA2-ON1
KX510260-KEN-10
KP317936-KEN-10-GA2-ON1
JX682762 CQ11-NA3
AF SX11-01NA3(10)
KP860084-NL-07
KP317926-KEN-07-GA2-ON1
JX015493-NL-08-GA2
JX015492-NL-07-GA2
KJ939966-VN-10-NA1
KF826830-GER-09
KF826831-GER-09
JF714709-KSA-8
KJ627328-PE-10
JX015482-BE-06-GA2
JF920051-USA-07-GA2
JX015499-BE-08-GA2
KJ627362-PE-09
KF826840-MEX-07
KJ627281-PE-09
JF920050-USA-809-GA2
KF826838-AR-06
JX015497-BE-08-GA2
JX015481-BE-09-GA2
KJ627290-PE-09
KP317950-KEN-09-GA2-ON1
KP317937-KEN-07-GA2-ON1
KP317933-KEN-08-GA2-ON1
JX015486-NL-05-GA2
AY114149-SG-02s-GA2/7
JX198128-USA-04-GA2
JQ901458-NL-03-GA2
JF920057-USA-98-GA2
JX198120-USA-04-GA2
KJ627311-PE-08
KJ627327-PE-07
KJ627324-PE-09
AY526550-RSA-01-GA2
KP317944-KEN-06-GA2-ON1
KF826848-AUS-07
JX015480-NL-07-GA2
JQ901453-NL-02-GA2
JQ901452-NL-01-GA2
KJ627352-PE-07
KU316141-USA-98
GU591770-USA-405
JX682745 CQ10-NA1(2)
AF BJ09-105NA4(4)
KP119747 GZ08(5)
AF BJ04-32GA2(4)
AF512538-SG-02s-GA2/7
KU950573-USA-06
AY526551-RSA-98-GA2
KU316131-USA-94
FJ948820-NL-99
AF BJ04-44GA2
KU316118-USA-96
AY198176 HB03(3)
KU316166-USA-77
KU316142-USA-82
KP258734-USA-89
KP258723-USA-86
AY526554-RSA-97-GA7
LN890642-UK-N/A-GA7
JF920065-USA-98-GA7
JX198132-USA-05-GA7
GU591771-USA-405
KU316178-USA-79
KU316149-USA-77
KJ723468-USA-88
JX198137-USA-87-GA3/SAA1
AY526557-RSA-98-SAA1
KU316150-USA-78
KU316174-USA-87
KP258730-USA-87
JX198113-USA-87-GA1
KP258695-USA-88
KU316107-USA-95
KU316099-USA-97
KU316109-USA-92
KJ723474-USA-89
KJ723478-USA-82
KU316103-USA-83
KJ723467-USA-86
KU316152-USA-85
KJ723475-USA-86
KU316112-USA-80
KU316171-USA-77
JX198136-USA-61-GA1
KJ155694-AUS-61
JX198138-AUS-61-A2
JF920069-USA-7-GA1
JX198112-USA-Long strain
KU316135-USA-81
KP258725-USA-80
KJ723462-USA-92
KU316104-USA-91
KJ723473-USA-90
KU316145-USA-96
JX198135-USA-94-GA5
KU316180-USA-98
KP258727-USA-97
AY526553-RSA-98-GA5
AF BJ09-55GA5
JX198127-USA-04-GA5
KF826847-AUS-07
JQ901455-NL-02-GA5
JQ901451-NL-01-GA5
KJ939948-VN-10-GA5
JX015485-NL-05-GA5
GU591762-USA-304
KF826854-ITA-09
KF530260-AR-05
JX069803-USA-00
AY526552-RSA-01-GA5
AY114151-SG-02s-GA2/7
KP317949-KEN-03-GA2-ON1
JQ901450-NL-01-GA5
JQ901448-NL-01-GA5
KF973329-USA-02
AF HN13-10GA5(2)
KF826846-AR-08
KF826836-MX-06
KF826828-AR-04
JX015487-NL-06-GA5
JX198117-USA-04-GA5
KU950479-USA-12
KF826826-MX-04
JX198143-CH18537 strain-Subgroup B
0.01
74
79
99
85
96
99
80
86
99
73
94
96
94
94
92
76
75
100
100
92
98
100
99
87
89
87
70
A11
A10
A9
A8
A7
A6
A5
A4
A3
A2
A1
A2

Supplementary Figure S2. Phylogenetic tree generated by maximum likelihood method of representative HRSVB F sequences from worldwide samples and Chinese samples from 1962 to 2014.

For representative Chinese sequences that have sequences identical or similar (nucleotide difference less than 2) to them, the total numbers of identical or similar sequences are shown within parentheses behind the names of the respective representative sequences.

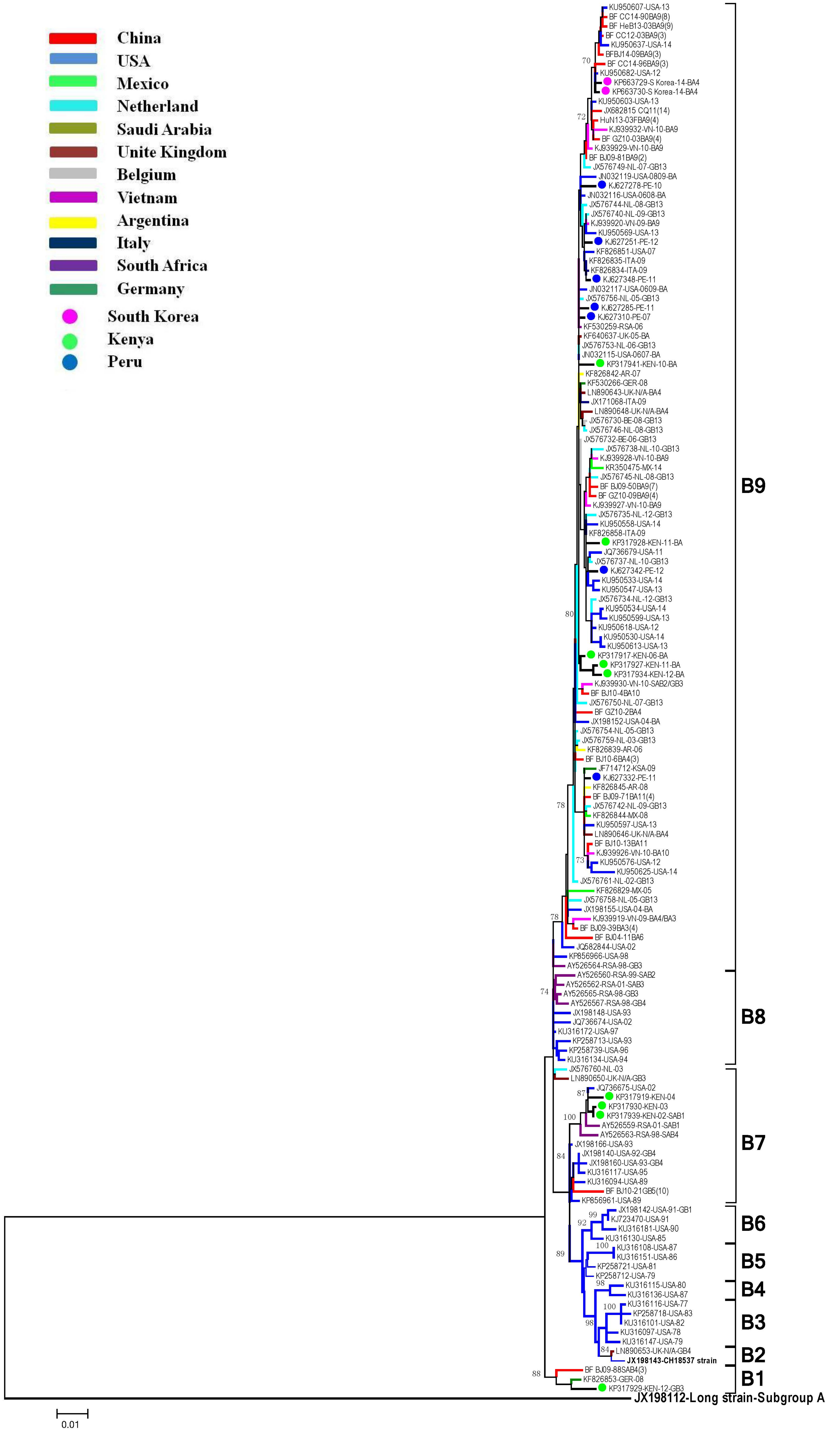
China
USA
Mexico
Netherland
Saudi Arabia
Unite Kingdom
Belgium
Vietnam
Argentina
Italy
South Africa
Germany
South Korea
Kenya
Peru
KU950607-USA-13
BF CC14-90BA9(8)
BF HeB13-03BA9(9)
BF CC12-03BA9(3)
KU950637-USA-14
BFBJ14-09BA9(3)
70
BF CC14-96BA9(3)
KU950682-USA-12
KP663729-S Korea-14-BA4
KP663730-S Korea-14-BA4
KU950603-USA-13
72
JX682815 CQ11(14)
HuN13-03FBA9(4)
KJ939932-VN-10-BA9
BF GZ10-03BA9(4)
KJ939929-VN-10-BA9
BF BJ09-81BA9(2)
JX576749-NL-07-GB13
JN032119-USA-0809-BA
KJ627278-PE-10
JN032116-USA-0608-BA
JX576744-NL-08-GB13
JX576740-NL-09-GB13
KJ939920-VN-09-BA9
KU950569-USA-13
KJ627251-PE-12
KF826851-USA-07
KF826835-ITA-09
KF826834-ITA-09
KJ627348-PE-11
JN032117-USA-0609-BA
JX576756-NL-05-GB13
KJ627285-PE-11
KJ627310-PE-07
KF530259-RSA-06
KF640637-UK-05-BA
JX576753-NL-06-GB13
JN032115-USA-0607-BA
KP317941-KEN-10-BA
KF826842-AR-07
KF530266-GER-08
LN890643-UK-N/A-BA4
JX171068-ITA-09
LN890648-UK-N/A-BA4
JX576730-BE-08-GB13
JX576746-NL-08-GB13
JX576732-BE-06-GB13
JX576738-NL-10-GB13
KJ939928-VN-10-BA9
KR350475-MX-14
JX576745-NL-08-GB13
BF BJ09-50BA9(7)
BF GZ10-09BA9(4)
KJ939927-VN-10-BA9
JX576735-NL-12-GB13
KU950558-USA-14
KF826858-ITA-09
KP317928-KEN-11-BA
JQ736679-USA-11
JX576737-NL-10-GB13
KJ627342-PE-12
KU950533-USA-14
KU950547-USA-13
JX576734-NL-12-GB13
80
KU950534-USA-14
KU950599-USA-13
KU950618-USA-12
KU950530-USA-14
KU950613-USA-13
KP317917-KEN-06-BA
KP317927-KEN-11-BA
KP317934-KEN-12-BA
KJ939930-VN-10-SAB2/GB3
BF BJ10-4BA10
JX576750-NL-07-GB13
BF GZ10-2BA4
JX198152-USA-04-BA
JX576754-NL-05-GB13
JX576759-NL-03-GB13
KF826839-AR-06
BF BJ10-6BA4(3)
JF714712-KSA-09
KJ627332-PE-11
KF826845-AR-08
BF BJ09-71BA11(4)
78
JX576742-NL-09-GB13
KF826844-MX-08
KU950597-USA-13
LN890646-UK-N/A-BA4
BF BJ10-13BA11
KJ939926-VN-10-BA10
73
KU950576-USA-12
KU950625-USA-14
JX576761-NL-02-GB13
KF826829-MX-05
JX576758-NL-05-GB13
JX198155-USA-04-BA
78
KJ939919-VN-09-BA4/BA3
BF BJ09-39BA3(4)
BF BJ04-11BA6
JQ582844-USA-02
KP856966-USA-98
AY526564-RSA-98-GB3
AY526560-RSA-99-SAB2
AY526562-RSA-01-SAB3
74
AY526565-RSA-98-GB3
AY526567-RSA-98-GB4
JX198148-USA-93
JQ736674-USA-02
KU316172-USA-97
KP258713-USA-93
KP258739-USA-96
KU316134-USA-94
JX576760-NL-03
LN890650-UK-N/A-GB3
JQ736675-USA-02
87
KP317919-KEN-04
KP317930-KEN-03
100
KP317939-KEN-02-SAB1
AY526559-RSA-01-SAB1
AY526563-RSA-98-SAB4
JX198166-USA-93
JX198140-USA-92-GB4
84
JX198160-USA-93-GB4
KU316117-USA-95
KU316094-USA-89
BF BJ10-21GB5(10)
KP856961-USA-89
JX198142-USA-91-GB1
99
KJ723470-USA-91
92
KU316181-USA-90
KU316130-USA-85
100
KU316108-USA-87
89
KU316151-USA-86
KP258721-USA-81
KP258712-USA-79
98
KU316115-USA-80
KU316136-USA-87
100
KU316116-USA-77
KP258718-USA-83
98
KU316101-USA-82
KU316097-USA-78
KU316147-USA-79
84
LN890653-UK-N/A-GB4
JX198143-CH18537 strain
88
BF BJ09-88SAB4(3)
KF826853-GER-08
KP317929-KEN-12-GB3
JX198112-Long strain-Subgroup A
B9
B8
B7
B6
B5
B4
B3
B2
B1
0.01
